# Iodine Absorption Cells Purity Testing

**DOI:** 10.3390/s17010102

**Published:** 2017-01-06

**Authors:** Jan Hrabina, Massimo Zucco, Charles Philippe, Tuan Minh Pham, Miroslava Holá, Ouali Acef, Josef Lazar, Ondřej Číp

**Affiliations:** 1 Institute of Scientific Instruments of the Czech Academy of Sciences, v.v.i., Královopolská 147, 61264 Brno, Czech Republic; tuan@isibrno.cz (T.M.P.); hola@isibrno.cz (M.H.); joe@isibrno.cz (J.L.); ocip@isibrno.cz (O.Č.); 2 National Institute of Metrological Research, Strada delle Cacce 91, 10135 Torino, Italy; m.zucco@inrim.it; 3 Sytèmes de Référence Temps Espace, SYRTE/CNRS-UMR 8630/LNE/Observatoire de Paris/UPMC, 61, Avenue de l’Observatoire, 75014 Paris, France; charles.philippe@obspm.fr (C.P.); ouali.acef@obspm.fr (O.A.)

**Keywords:** iodine cells, absorption spectroscopy, laser spectroscopy, laser standards, frequency stability

## Abstract

This article deals with the evaluation of the chemical purity of iodine-filled absorption cells and the optical frequency references used for the frequency locking of laser standards. We summarize the recent trends and progress in absorption cell technology and we focus on methods for iodine cell purity testing. We compare two independent experimental systems based on the laser-induced fluorescence method, showing an improvement of measurement uncertainty by introducing a compensation system reducing unwanted influences. We show the advantages of this technique, which is relatively simple and does not require extensive hardware equipment. As an alternative to the traditionally used methods we propose an approach of hyperfine transitions’ spectral linewidth measurement. The key characteristic of this method is demonstrated on a set of testing iodine cells. The relationship between laser-induced fluorescence and transition linewidth methods will be presented as well as a summary of the advantages and disadvantages of the proposed technique (in comparison with traditional measurement approaches).

## 1. Introduction

The metrology of length and time is currently developing fast. New approaches exploring quantum effects on single atoms or ions are being introduced [1,2,3,4]. While the definition of the second will most likely stay unchanged for some time (thanks to the significant improvement of the Cesium atomic clock), the unification of the fundamental metrology of time and length can be expected in the future. An ultra-highly stable oscillator operating on an optical frequency with a coherent down-conversion to the radiofrequency range through femtosecond optical frequency synthesis will comprise both requirements [5,6].

Optical frequency standards based on traditional spectroscopy in gaseous media may lose the status of the primary standards in the future. However, the complexity and price of optical clocks ensure their further utilization. There are numerous applications where the robustness, simplicity, cost and reliability together with a compact design play a key role. Examples of that are systems designed for airborne or even space-borne applications [7], optical telecommunications [8] or lasers for ballistic gravimeters [9].

The conventional laser standards based on locking of the laser frequency to the transitions in gas absorption cells offer very good spectral properties with relative frequency stabilities down to 1 × 10^−14^ levels [10,11,12]. These stabilities are for sure sufficient for many scientific purposes covering high-resolution laser interferometry and precise dimensional measurement, auxiliary laser sources for experiments in physics such as atom cooling and atomic and ion clocks, optical telecommunications, optical sensors and others [8,13,14,15]. The practical implementation of the optical frequency reference has the form of a glass tube/absorption cell, the spectral parameters of which fundamentally determine the overall frequency stability of the implemented stabilization scheme [16].

Molecular iodine is one of the most common absorption media in the technology of gas cell stabilized lasers. This medium offers a rich set of strong and very narrow absorption lines which cover a significant part of the visible spectra (approximately from 500 to 700 nm) [17,18,19]. The iodine transitions overlap with many laser wavelengths, and thanks to these features this medium has been selected and recommended as an optical frequency reference for the practical implementation of the international system of units (meter) at several wavelengths [10]. Today most stable conventional laser standards (the frequency doubled Nd:YAG laser with a 532 nm wavelength) stabilized for iodine transition can achieve or even break the relative frequency stability level of 2 × 10^−14^ at 1 s integration times [7].

The main disadvantages of the molecular iodine used as an optical frequency reference are its high corrosivity in contact with many materials (especially metals) and its enormous sensitivity to the presence of chemical impurities and foreign molecules in the absorption media. This contamination causes frequency shifts of absorption spectral components, broadening of absorption lines and, overall, an effect of degradation of the achievable frequency stability and accuracy of the implemented laser standard. Due to these reasons the chemical purity and spectral properties of the iodine absorption cells must be precisely evaluated and controlled [16]. From the point of approximate iodine cells’ purity qualification, they can be divided into three purity levels: good purity with a Stern-Volmer coefficient (laser-induced fluorescence measurement method) *K*_0_ < 1 Pa, suitable for the most precise laser standards with minimal frequency shifts; middle-polluted cells with *K*_0_ = 1~2 Pa, suitable for less demanding applications such as linear absorption spectroscopy stabilization systems; and highly contaminated cells with *K*_0_ > 2 Pa (inappropriate for further usage). Their differentiation will be further discussed in Section 3 and Section 4 [20].

In this paper, techniques for the iodine cell quality measurement and evaluation will be compared and discussed with a brief background dealing with the various iodine cell manufacturing and preparation technologies. The results presented in this article build on the experience and results of the three laboratories of INRIM (National Institute of Metrological Research, Torino, Italy), LNE-SYRTE (Laboratoire National de Métrologie et d’Essais, Time-Space Reference Systems, Paris, France) and ISI (Institute of Scientific Instruments, Czech Academy of Sciences, Brno, Czech Republic). Three methods are considered, including laser-induced fluorescence, absolute frequency measurement and linewidth measurement. Their signal-to-noise performance contributing to uncertainty is presented, together with physical, technical and systematic limitations the methods face. In the end, we offer the conversion relationship between them as well. The results are presented on quite various sets of cells made at two of the three institutions. Cells differing in glass material, shape, dimensions, window quality and the technology of filling are, on one hand, more difficult to compare, but, on the other, they show the limits of the reproducibility of the measuring methods and their sensitivity to the configuration of the setup quite well. Manufacturing and quality testing of the cells has to follow the needs of metrology practice and this information especially we consider very important for the metrology of highly stable optical frequencies.

## 2. Absorption Cell Technology

Iodine absorption cells are usually made of fused silica glass (quartz), a material which does not interact with the iodine gas and allows the preservation of its high chemical purity. Quartz optical windows ensure a high optical quality of surfaces (important for the suppression of laser beam wavefront distortion) and, moreover, they can be equipped with optical coatings (antireflective—AR, high reflective—HR, or their combinations). The coatings can be deposited at the outer and also the inner surface of the windows; for the visible spectral range they are usually made of layers of TiO_2_ and SiO_2_, where the SiO_2_ layer is the top (covering) one. This approach ensures a non-contaminating inert environment for the iodine. In order for the cell to achieve the best possible chemical purity, a vacuum-tight connection of optical windows to the cell body must be accomplished without the use of any glue, solder or any other chemical substance. Due to this, two alternative methods were used for connecting the windows—high-temperature welding of the windows to the cell body/tube or a technology of optical contact. Requirements for the compactness of the laser standard systems lead to new approaches in the absorption cell technology such as multi-pass arrangement of the cell. This leads to a combination of AR and HR coatings allowing multiplying the laser beam path inside the cell [21]. The stabilized laser systems based on these cells can achieve ultra-high-frequency stabilities in very compact arrangements [7]. Another recent concept in absorption cell technology is the usage of hollow-core photonic crystal fibers (HC-PCF) as a cell body instead of the glass tubes. This approach brings a further reduction of the system weight and dimensions. Unfortunately, achievable spectral parameters and corresponding laser stabilities are usually limited by transit time and collisional broadening processes (small diameters of the fiber core structures); however, the technology of proper filling and sealing of the prepared fiber cells is still in progress and under development [22,23,24].

A crucial step in iodine cell technology is the preparation of chemically clean absorption media. Iodine’s extreme corrosivity and sensitivity to the presence of impurities lead to the prerequisite of proper purification of the absorption media. The iodine has to be thoroughly purified in several steps with the help of molecular sieves and distillation processes, and the cell body has to be precisely cleaned and evacuated before filling with the absorption medium [21].

It is not always necessary to achieve the best possible laser source stability at all costs and less demanding applications (i.e., linear absorption spectroscopy setups) may be appropriate to utilize. In these cases, a simpler technology of cell manufacturing can be used. This approach includes the use of borosilicate glass tubes instead of fused silica as the cell body material, and float glass for the windows (reduced cost and easier manufacturing due to lower welding temperatures) or filling the absorption media to a certain/predefined level of iodine pressure (“starved” cells). If these cells are operated in environmental conditions of higher temperature than the corresponding iodine saturation point, they do not need external control of the iodine pressure (commonly stabilized by the temperature of the cell cold finger with the presence of solid iodine and an external Peltier cooler), although in terms of the stability of the laser standard, they still can achieve very good performances [21,25].

## 3. Measurement of Iodine Cell Purity

The spectral properties and chemical purity of the iodine cells fundamentally affect the resulting parameters of the iodine-stabilized lasers and therefore they must be not only precisely controlled but also evaluated.

This verification can be done through several techniques, but unfortunately all of them carry some disadvantages and limitations. Thus, we are currently looking for new methods for iodine cell purity evaluation.

### 3.1. Laser-Induced Fluorescence (LIF) Method

One of the traditionally used methods for the contamination inspection of iodine cells is the method of laser-induced fluorescence. The iodine cell is irradiated by laser light and the level of the induced fluorescence is detected. The presence of impurities in absorption media causes collisional quenching, a relaxation process of excited iodine molecules evoked by collisions with atoms and molecules of impurities. This process reduces the lifetime of the excited state, causes non-radiative transitions of iodine to the fundamental level and therefore reduces the level of induced fluorescence. The relative intensities of induced fluorescence at selected iodine pressure levels are measured and the results are processed by the normalized Stern-Volmer equation (Equation (1)) [26]:

(1) $$\frac{I_{0}}{I_{F}}=K_{0}\frac{1}{p_{I}}+L_{0}$$

where *I_F_* is the fluorescence intensity, *I*_0_ is the normalizing parameter (in our case the fluorescence intensity at 10 Pa), *p_I_* is the iodine pressure, *L*_0_ is the factor depending on the molecule parameters and, finally, *K*_0_ (the Stern-Volmer coefficient) is the parameter dependent on the foreign gas partial pressure, through the approximate equation (Equation (2)):

(2) $$K_{0}=A+B\sigma_{X}v_{X}p_{X}$$

where *A* and *B* are parameters dependent on the first order of the iodine parameters, *σ_x_* is the collision cross-section between the iodine molecule and the foreign gas molecule, *v_x_* is the mean relative velocity and *p_x_* is the partial pressure of the foreign gas. As the *K*_0_ is directly proportional to the changes of *p_x_*, the iodine cells can be compared among each other without knowing the cross-sections involved, simply by comparing their *K*_0_ values. The Stern-Volmer coefficient is the slope of the relation between the iodine pressure and the detected fluorescence intensity and it expresses the purity of the iodine cell [20,27]. Although this method is based on a relatively simple experimental setup, it carries several disadvantages. It usually uses a quite rare and noisy argon-ion laser source with a 502 nm wavelength, where the collisional quenching process has an acceptable sensitivity (long lifetime of the excited state); nonetheless, it requires a photomultiplier fluorescence detection sensitive to the presence of stray and background light (it is not very suitable for cells without AR-coated or Brewster angled optical windows). As shown in [20,27], this method strikes the physical-technical resolution limit/saturation floor especially in the case of very clean (good purity, *K*_0_ < 1 Pa levels) iodine cells.

### 3.2. Absolute Frequency Measurement

The second well-known method for iodine cell purity testing is the absolute optical frequency measurement of the iodine-stabilized laser (locked to selected iodine transition in the cell under test). The presence of impurities in the absorption medium causes a frequency shift of the transition from its theoretical value with a direction and intensity which depend on the impurity molecule specie. This method requires precise stabilization of the laser to the selected hyperfine iodine transition (for example, by the saturated absorption spectroscopy method) and measurement of the laser absolute frequency by the beat-note measurement with the help of some reference (stabilized frequency comb, reference iodine-stabilized laser) [13,28,29]. The main disadvantages of this technique are the requirement of a relatively complicated experimental setup and its proper adjustment, limitations for the measurement of highly contaminated cells (loss of signal-to-noise ratio) and also the critical influence of a large number of parameters that are difficult to preserve and have their repeatability ensured (frequency shifts affected by the lens effect, wavefront distortion, external magnetic field) [20,30].

### 3.3. Hyperfine Transition Linewidth Measurement

Considering the disadvantages of the traditional methods mentioned above, we have recently introduced an alternative technique for the inspection of iodine cells based on hyperfine transition linewidth measurement [21]. The linewidth of the iodine transition is strongly dependent on the level of contamination, which causes collisional broadening effects of the absorption line and the reduction of the iodine molecules’ excited-state lifetime. The method uses precise scanning and recording of the iodine hyperfine spectrum with the help of tuning the laser frequency by a digitally controlled acousto-optical frequency shifter, the saturated absorption spectroscopy method with the detection of the hyperfine signal by third harmonics (3f method), followed by the post-processing of obtained data by the inverse 3f algorithm to receive the original Lorentzian lineshape of the investigated transition [31,32]. The experimental setup was previously used for the scanning of the undocumented part of the iodine spectra within a project that was oriented to the iodine-stabilized frequency-tripled fiber laser. The main advantages of this method are its relative simplicity, the possibility of the measurement of cells with different designs, the chance to perform measurements at different wavelengths/transitions (considering the different natural linewidths for different lines), and good sensitivity to the contamination of the iodine medium; furthermore, this method does not suffer from the limitations of high-purity iodine cell measurement of the frequency shifts and induced fluorescence methods mentioned above. On the other hand, the method is not very suitable for highly polluted cells, where the signal-to-noise ratio limits the detection of the hyperfine profile. The experimental arrangement should also ensure the sufficiently large diameter of the laser beam to exclude the hyperfine transition spectral linewidth broadening due to the time-of-flight effect.

The testing of the chemical purity of iodine absorption cells is a complex task which requires the selection of proper evaluation methods depending on the cell design and iodine purity (contamination level) class. This article deals with the analysis and comparison of all of the above-mentioned methods and summarizes their advantages and disadvantages based on the results obtained from the measurement of testing sets of iodine cells with different parameters and designs. The article gives the recommendation for the selection and practical usage of the iodine cell purity evaluation methods and it explains the results of their quantitative relationships.

## 4. Experimental Results

### 4.1. A Set of Testing Iodine Cells

The experimental work of the characterization of purity evaluation methods of iodine cells was based on the testing a set of iodine cells. This set covers different designs, iodine contamination levels and the final purpose of the cells to have an opportunity to compare the suitability of the measurement methods to each other (Table 1). The cells C1–C2 are made of a borosilicate glass, are filled with a certain pressure of iodine vapor (with a corresponding saturation temperature point of +14 °C) and are intended for usage in less demanding applications such as linear absorption spectroscopy stabilization setups. The cells C3–C8 and C10 were prepared and intended as references for high-precision laser standards, have quasi-parallel windows (C3–C6 and C10 are further equipped with antireflection coatings (AR) for 532 and 633 nm wavelengths) and are made of fused silica glass. C10 was previously recognized as a highly contaminated cell with a leakage of absorption medium and was selected for the testing set for the purpose of analyzing individual methods. C9 is intended for the intra-cavity arrangement of a 633 nm He-Ne/I_2_ laser standard and it is equipped with Brewster angled optical windows. All of the cells were manufactured/filled during the 2003–2014 period.

### 4.2. Laser-Induced Fluorescence Systems Comparison and Improvement

**Figure 1:**
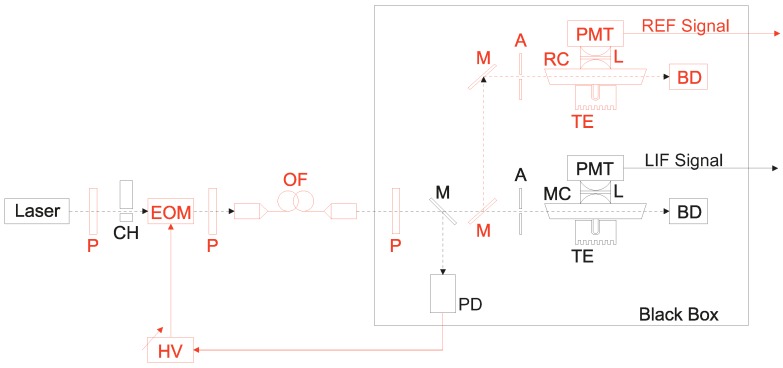
Comparison of original (black color) and upgraded (black + red colors) laser-induced fluorescence experimental setups. The upgrade includes active stabilization of the excitation laser intensity with the help of the electro-optical amplitude modulator (EOM), the monitoring of the spectral condition of the laser source by the reference cell (RC) and the implementation of correction for backscattered light by the procedure of cooling the cell with liquid nitrogen. Laser—502 nm Argonion laser, P—polarizers, CH—optical chopper, HV—high voltage source and PID regulator, PD—photodetector of laser intensity, OF—optical fiber, M—mirrors, A—apertures/irises, PMT—photomultipliers, L—collimation optics/lenses, TE—temperature control of cells iodine pressure, BD—beam dumps/traps, RC—reference iodine cell, MC—measured iodine cell, Black Box—box made of light-absorptive material to suppress the back reflections influences.

**Figure 2:**
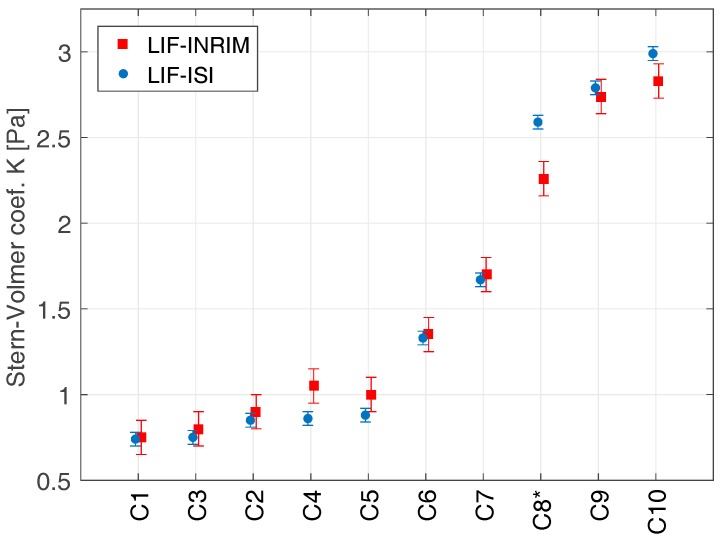
Comparison of the measurements of the original (INRIM) and the upgraded (ISI) LIF setups performed on the same 10 pcs iodine cell set. * The progress in C8’s iodine purity degradation is probably caused by a small leakage on the cell body (release of contaminants).

A characterization of limits and aspects of the laser-induced fluorescence method (LIF) was done by comparison of two independent experimental setups on the same set of iodine cells. The original LIF setup [27] (operated at INRIM, National Institute of Metrological Research, Torino, Italy) was compared to the second system (operated at ISI, Institute of Scientific Instruments, Czech Academy of Sciences, Brno, Czech Republic) equipped and improved with a compensation system suppressing the laser power and spectral noise. The schema of the measurement setups with the highlighted improvements is shown in Figure 1. The light from the Ar-ion laser (*λ* = 502 nm, *P_OUT_* ~ 5 mW, ~5 GHz linewidth) passes through an optical chopper (CH, running at 500 Hz frequency) and excites iodine molecules in the measured cell (MC). The level of induced fluorescence is monitored by the photomultiplier (PMT) and processed by synchronous detection (driven by the same 500 Hz source as the optical chopper). The pressure of the iodine medium is controlled by the Peltier cooler with the digital temperature driver (TE, mK level stability and accuracy). Measuring part of the optical setup with the cell and the photomultiplier is placed inside a box covered with light-absorptive material to minimize the scattered light influence of the detection. The laser light passed through the cell is fed into the non-reflecting beam dump (BD). The improvement of the original setup is represented by (1) the inclusion of the active stabilization of the laser source intensity; (2) the addition of the reference iodine cell for monitoring of the laser source frequency drift and mode-hops and (3) the correction for the backscattered light-associated errors. The power drift of the used Ar-ion laser was compensated by the driving of the electro-optical amplitude modulator (EOM) controlled by the synchronously demodulated signal from the auxiliary photodetector (PD, 10 kHz bandwidth) processed by the lock-in amplifier (referenced again by the signal from the optical chopper, *f_REF_* = 500 Hz). As the laser source suffered spectral instability, which directly influenced the level of the detected fluorescence (varying coincidence with the proper iodine transition R(26) (62-0)), we improved the setup with a reference iodine cell (RC), and a corresponding detection part with the second photomultiplier (PMT), whose iodine pressure was held at a constant value, and the detected fluorescence signal operated as a monitor of the laser spectral stability. The data from the reference cell was used as a normalizing parameter in the measured cell fluorescence level processing. The level of stray light and background scattered radiation was measured just after the cell was mounted into the setup and before the measurement of the Stern-Volmer coefficient. The iodine pressure was reduced to a negligible level with the help of cooling the cell cold finger with liquid nitrogen (LN_2_), and after a few minutes when all of the iodine became trapped in a solid state, the level of the background light was recorded. This value was then used for LIF data correction (subtracted from the measured fluorescence level) during the next LIF measurement. This scattered light level detection was conducted for both the measured and the reference cells. All of the cells were measured both by the INRIM setup and by the ISI (improved design) experimental setup, covering iodine pressure ranges between 2 and 10 Pa. Corresponding LIF data (computed Stern-Volmer coefficients) obtained from both independent systems are recorded in Table 2 and Figure 2. They show a very good agreement which covers the expected reproducibility uncertainty of the methods (estimated pooled standard uncertainty of the INRIM setup of 0.2 Pa, *k* = 2, confidence level of 95%) [20]. The discrepancy between LIF values for the “C8” cell was possibly caused by the long-term increase of the amount of impurities in the iodine due to a miniature leakage in the cell body (corresponding INRIM values for C7–C9 cells were measured in 2010, ISI values in 2014). The comparison confirms a significant improvement of the experimental setup, even though the Ar-ion laser used in the ISI arrangement performed with a worse spectral and intensity stability. Moreover, repeated measurements confirmed that although these improvements represent an increase of the hardware complexity of the LIF method experimental setup, they also carry a significant improvement of the Stern-Volmer *K*_0_ measurement reproducibility down to 0.04 Pa (*k* = 2). For them to stay correlated to the previous INRIM work [20], the reproducibility of the method was estimated as a pooled standard deviation of the repeated measurement of 18 iodine cells with different iodine contamination levels by (Equation (3)):

(3) $$S=\sqrt{\frac{\left(n_{1}-1\right)S_{1}^{2}+\left(n_{2}-1\right)S_{2}^{2}+\ldots +\left(n_{k}-1\right)S_{k}^{2}}{\left(n_{1}-1\right)+\left(n_{2}-1\right)+\ldots +\left(n_{k}-1\right)}}$$

where $n_{1}$, $n_{2}$, …, $n_{k}$ are the sizes of the LIF data subsets for each measured cell and $S_{1}^{2}$, $S_{2}^{2}$, …, $S_{k}^{2}$ are their respective variances.

### 4.3. Transition Linewidth versus Laser-Induced Fluorescence Methods

The hyperfine transition linewidth measurement was performed using an experimental system based on a frequency-doubled Nd:YAG laser at a 532 nm wavelength and the saturated absorption spectroscopy setup (modulation frequency of 1.04 kHz, modulation depth of 800 kHz, detection by the 3f technique [31,33]) described in [21]. The selected cells’ evaluation was processed on the well-investigated and known ^127^I_2_ R(56) (32-0) line and its hyperfine transition component *a*_10_ (recommended and often used for the realization of 532 nm iodine-stabilized laser standards). The laser frequency was locked with the help of the reference cell to the hyperfine component *a*_6_ (−170.064 MHz far from *a*_10_) and the *a*_10_ spectral profile scan was done by tuning of the AOM modulator/shifter (in double-pass configuration) of 85 ± 2 MHz. Each cell was measured several times for different iodine media pressures (0.8–4.14 Pa), different saturation power intensities (2.3–32 mW/cm^2^), and finally zero-pressure and zero-power broadened linewidth values were calculated by the inverse 3f algorithm [21].

**Figure 3:**
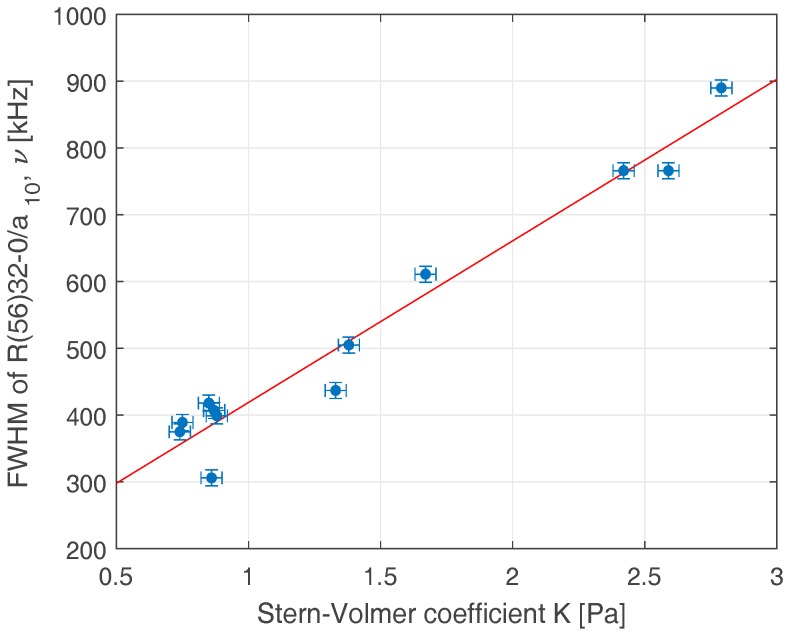
Relationship between laser-induced fluorescence (ISI LIF setup results) and hyperfine transition linewidth measurements (532 nm wavelength line R(56) (32-0), component *a*_10_).

To find a relationship between iodine cells’ hyperfine transition linewidths and a comprehensive database of previous LIF measurements of many of iodine cells made at INRIM [20], we decided to compare the linewidth method values to the LIF results of the same set of cells. The link between these methods can help to characterize better the assumed frequency stability of the iodine-locked laser standard with the aim to select a proper iodine optical frequency reference for the intended system. The measurement results and relation between the LIF (Stern-Volmer coefficient *K*_0_ measured by the ISI upgraded setup) and natural linewidth measurements (*Δν*) for 12 different iodine cells are shown in Figure 3. The corresponding interdependence slope valid for the selected hyperfine transition (R(56) (32-0), *a*_10_) obtained by the linear regression fit of recorded data is *Δν*/*K*_0_ = 242 kHz/Pa with a 177 kHz offset, and the computed correlation coefficient is 0.978. The *Y*-axis error bars express the estimated uncertainty of the linewidth measurement (12 kHz, *k* = 2) obtained by the computation of the pooled standard deviation from the results of the measured 12 cells and the reflected confidence level of 95%.

The results show a comparable level of the estimated measurement uncertainty between the linewidth and improved LIF methods, fully confirming the usefulness of the proposed linewidth technique for the inspection of iodine cell purity. Moreover, as the LIF method is constrained by its limits, described in Section 3.1, the linewidth method can be advantageously used for the inspection of high-purity iodine cells. Considering the uncertainty estimates of both methods and the slope of the relation in Figure 3, the reproducibility of the linewidth method expressed in LIF (*K*_0_) values corresponds to: $\mu_{\vartheta }=\pm 0.0495\;\mathrm{Pa}$.

Moreover, considering previous work and results published in [20], where dependencies between absolute frequency shifts (*Δf*) and induced fluorescence (*ΔK*_0_) methods were studied in detail, we can follow these data to find the relation between shift and linewidth measurements. From [20], the *Δf*/*ΔK*_0_ ratio (for the same iodine transition *a*_10_ of the R(56) (32-0) line, observed with the modulation transfer technique) gives *Δf*/*Δ*K_0_ = (−3.4 ± 0.6) kHz/Pa, which corresponds to the linewidth/shifts relation of Equation (4):

(4) $$\Delta \vartheta =\left(177\pm 12\right)-\left(71\pm 13\right)\times \Delta f_{532}\;\left(\mathrm{kHz};\mathrm{kHz}\right)$$

where (177 ±12) (kHz) expresses the offset and reproducibility of the linewidth method and $-\left(71\pm 13\right)\times \Delta f_{532}\;\left(\mathrm{kHz};\mathrm{kHz}\right)$ shows the relation slope between shift and linewidth methods.

The linewidth method can be also advantageously applied for the adequate evaluation of hollow-core photonic crystal fiber (HC-PCF) based optical frequency references, where the obtained results can inform not only the cell purity, but the method can additionally serve as an inspection of the overall cell’s spectral properties (analysis of influences of overall transition linewidth—transit-time broadening, power broadening, and collisional broadening effects). Furthermore, it can help with an estimation of the expected frequency stability of the intended fiber-based optical frequency reference-stabilized laser.

### 4.4. Recommendation for the Purity Evaluation of Iodine Absorption Cells

Summarizing all of the achieved results, the recommendations for the proper method selection for the purity evaluation of iodine cells, including the main advantages and limitations, are shown in Table 3.

The method of induced fluorescence benefits from a relatively simple opto-mechanical setup and quick measurement without complicated adjustments. The main disadvantages of this method can be seen in the necessity of a quite rare laser wavelength (in our case ~502 nm, where the collisional quenching process is sensitive enough), problems with stray and backscattered light suppression for short and non-standard-design cells, the impossibility of measuring fiber-based cells (filled HC-PCF fibers), the need for a reference cell in the case of using the improved LIF setup and also in it reaching its resolution limits in the case of very clean cells (*K*_0_ < 1 Pa, need for precise adjustment of the setup, especially perfect suppression of the stray light influence and the monitoring of laser spectral properties/mode-hops). This method can be recommended especially for middle- to highly contaminated cells, where the measurement can be done quickly and easily while ensuring a very good reproducibility.

The proposed method of hyperfine transition linewidth measurement has advantages which can be summarized as follows: it has a good sensitivity and resolution, it can use existing laser standard setups, it can be performed at different available laser wavelengths (where the signal-to-noise ratio of the iodine hyperfine transition is good enough) and it is also suitable for the measurement of fiber-based optical frequency references (filled HC-PCF), where it can serve as an overall inspection method of the reference properties. The drawbacks are: degradation of the signal-to-noise ratio for highly polluted cells, and the need for precise adjustment of the optical setup and sophisticated processing (time requirements). Because of the above properties, this method is suitable for the inspection of very clean and middle-contaminated iodine cells (*K*_0_ < 2 Pa).

The traditional method of absolute frequency shift measurement can use existing laser standard setups and the direction and intensity of the shift can give an indication of the contaminant type [34]. However, the reproducibility of the measurement of absolute frequencies varies strongly with the selected type of laser standard and wavelength, which can make the proper characterization of the iodine cell purity more difficult [20]. The main disadvantages of this technique can be seen in the degradation of the signal-to-noise ratio for highly contaminated cells, the need for precise control of many of overall shift-affecting parameters (lens effect, power/pressure broadening, transit time, wavefront distortion for different opto-mechanical arrangements and different designs of the cells), the need for a precise (in the ideal case absolute) optical frequency reference for performing a beat-note measurement, as well as the inappropriateness of the method for the inspection of fiber-based cells (the achievable frequency stability is typically limited by the high transit-time broadening effect due to the narrow optical mode of the fiber).

### 4.5. Frequency Stability of Iodine-Stabilized Fiber Laser

**Figure 4:**
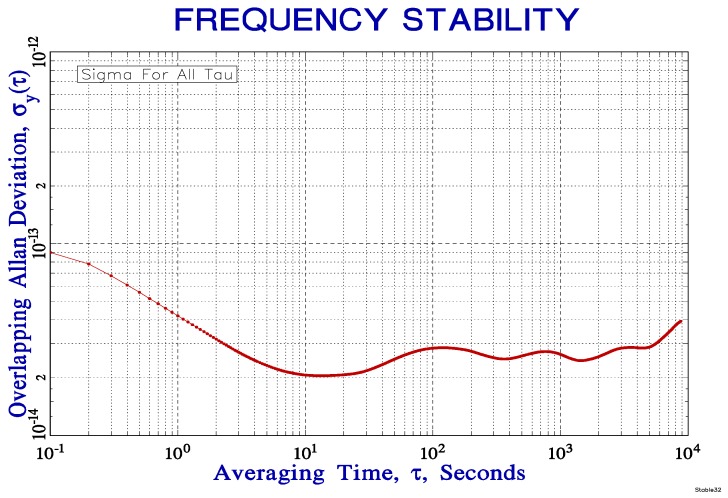
Measurement of the relative frequency stability (Allan deviation) of frequency-tripled fiber laser stabilized by “starved” type of borosilicate glass made iodine cell.

Thanks to excellent obtained parameters of the tested starved-type borosilicate cells (measured through both the linewidth and LIF methods), we decided to put these cells into the saturated spectroscopy laser stabilization system and measure the achievable frequency stability of the laser locked to these cells. This frequency stability measurement can test the potential progress of the contamination of iodine cells over the years. The stabilization scheme was based on an Erbium-doped fiber laser supported by an Erbium-doped fiber amplifier which gives about 800 mW at 1.54 μm and an all-fiber optical tripling stage (two cascaded periodically poled Lithium Niobate (PPLN) ridge nonlinear crystals in a single-pass configuration) with 290 mW of optical power in the green range (*λ* ~ 514 nm) at the output (conversion efficiency > 36%) [35]. The small part of this green laser light was fed into a classic modulation transfer spectroscopy setup [33,36] and the laser frequency was locked to detect the hyperfine transition *a*_1_ of the R(34) (44-0) line in the tested iodine cell (double-pass arrangement). The absolute value of the fundamental IR range laser frequency was detected by the beat-note measurement with the stabilized optical frequency comb [37]. The comb is referenced by an ultra-stable cylindrical optical cavity made of ULE (ultra-low-expansion glass) with a fractional frequency stability at the 1 × 10^−15^ level order, well below the expected stability of the measured iodine-stabilized laser [38]. The relative frequency stability was measured in both “starved” (without cold finger cooling) and also standard cell regimes to detect the possible impact of the precise iodine pressure control. With this arrangement we were able to achieve comfortably a short-term relative frequency stability at the level of 4 × 10^−14^ (for 1 s integration times) and keep the long-term stability value below this level up to 10^4^ s integration times for both the starved and common regimes of the cell (Figure 4). These results show not only a very high potential of these low-cost, more easily manufactured and easier-to-operate cells for the frequency locking of the laser standards, but also they confirm the negligible contamination of these borosilicate glass made iodine cells after more than three years after their filling.

## 5. Conclusions

We investigated the key properties and limits of possible measurement methods for the purity evaluation of iodine absorption cells and summarized the recommendations for testing these iodine cells. We selected a set of iodine cells with different optomechanical designs and absorption media purities and used them for detailed analysis of laser-induced fluorescence (LIF) and introduced spectral linewidth measurement techniques. The LIF method measurement limits were tested by comparison of the INRIM setup and the improved (ISI) experimental setups. This modification, which implements intensity stabilization of the incident laser power, compensation of backscattered light and reference cell detection of spectral properties of the laser, showed an important improvement in terms of the reproducibility of the measurement (0.04 Pa, approximately five times lower in comparison to the original system’s reproducibility estimation). Moreover, this measurement also confirmed the possibility of using a multimode excitation laser and the simplicity of the method. The results from the LIF measurements were compared to data received from the proposed method of hyperfine profile spectral linewidth measurement, where the same set of cells was used for performing the tests. These results were used for finding the limitations and advantages of these methods and for considering the practical recommendations for the purity evaluation of iodine cells. Next, the measurements confirmed the great chemical purity of starved-type iodine cells made of borosilicate glass instead of fused silica. The tested relative frequency stability of the cell-locked frequency-tripled fiber laser (in both starved and usual saturated regimes of operation of the references) showed an important potential of this low-cost cell variant.

## Figures and Tables

**Table 1:** Description of tested iodine absorption cells.

Cell Names	Date of Filling	Length (mm)	Active Diameter (mm)	Material, Comments
C1, C2	2013	400	20	Borosilicate, Starved to +14 °C
C3–C6	2014	500	22, AR coatings	Fused silica
C7	2003	500	20	Fused silica
C8	2003	300	22	Fused silica
C9	2010	180	10, Brewster	Fused silica, Brewster windows
C10	2013	300	22, AR coatings	Fused silica, Leakage/contaminated

**Table 2:** Comparison of the measurements of the original (INRIM) and upgraded (ISI) LIF setups performed on the same 10 pieces iodine cell set. * The progress in C8’s iodine purity degradation was probably caused by a small leakage on the cell body (release of contaminants during the four-year-long period).

Cell Name	C1	C3	C2	C4	C5	C6	C7	C8 *	C9	C10
LIF-INRIM (Pa)	0.75	0.80	0.90	1.05	1.00	1.35	1.70	2.26	2.74	2.83
Date of meas	2014	2014	2014	2014	2014	2014	2013	2011	2013	2013
LIF-ISI (Pa)	0.75	0.75	0.85	0.86	0.88	1.33	1.67	2.59	2.79	2.99
Date of meas	2014	2014	2014	2014	2014	2014	2014	2014	2014	2014

**Table 3:** Recommendations of the laser-induced fluorescence, hyperfine transition linewidth measurement and absolute frequency shifts measurement methods for the evaluation of iodine absorption cells’ purity. The relationships between the methods are valid for R(56) (32-0), *a*_10_ iodine hyperfine transition component.

	Laser-Induced Fluorescence Method	Hyperfine Transition Linewidth Method	Absolute Frequency Shifts Measurement
Advantages	Simple setup, quick measurement, easy adjustment	Good sensitivity, can use existing laser standard setup, can be used for HC-PCF based references evaluation, can be performed at different available laser wavelengths	Can use existing laser standard setups usually available in metrological labs. The direction and intensity of the shift could give an indication of the impurity specie.
Difficulties	Rare laser wavelength (502 nm), resolution limit for very good cells, particularly problems of stray light (for example short cells), impossible for HC-PCF based references evaluation, need a reference cell (in case of improved arrangement)	Problems for highly contaminated cells (SNR degradation), time requirements due to demanding measurement process	Problems for highly polluted cells (SNR degradation), many overall shift-affecting parameters (worse reproducibility for different opto-mechanical arrangement changes), need of additional absolute optical frequency reference, unadvisable for HC-PCF based reference testing (insufficient stability)
Suitable for	Middle-to-high polluted cells (*K*_0_ > 1 Pa)	Clean and middle-polluted cells (*K*_0_ < 2 Pa)	Clean and middle-polluted cells with possible locking of the laser (*K*_0_ < 2 Pa)
Relation slopes referenced to LIF method *		$\Delta \vartheta =177+\left(242\pm 12\right)\times K_{0}$ (Pa; kHz)	$\Delta f=\left(-3.4\pm 0.6\right)\times K_{0}$ (Pa; kHz)
